# AAMP is a binding partner of costimulatory human B7-H3

**DOI:** 10.1093/noajnl/vdac098

**Published:** 2022-06-30

**Authors:** Sara Ciprut, Anne Berberich, Maximilian Knoll, Stefan Pusch, Dirk Hoffmann, Jennifer Furkel, Aoife Ward Gahlawat, Lena Kahlert-Konzelamnn, Felix Sahm, Uwe Warnken, Martin Winter, Martina Schnölzer, Sonja Pusch, Andreas von Deimling, Amir Abdollahi, Wolfgang Wick, Dieter Lemke

**Affiliations:** Clinical Cooperation Unit Neurooncology, German Cancer Consortium (DKTK), German Cancer Research Center (DKFZ), Heidelberg, Germany; Department of Neurology, University of Heidelberg Medical School and National Center for Tumor Diseases (NCT), Heidelberg, Germany; Clinical Cooperation Unit Neurooncology, German Cancer Consortium (DKTK), German Cancer Research Center (DKFZ), Heidelberg, Germany; Department of Neurology, University of Heidelberg Medical School and National Center for Tumor Diseases (NCT), Heidelberg, Germany; Clinical Cooperation Unit Translational Radiation Oncology, German Cancer Consortium (DKTK) Core Center Heidelberg, German Cancer Research Center (DKFZ), Heidelberg, Germany; Division of Molecular and Translational Radiation Oncology, Department of Radiation Oncology, Heidelberg Faculty of Medicine (MFHD) and Heidelberg University Hospital (UKHD), Heidelberg Ion-Beam Therapy Center (HIT), National Center for Tumor Diseases (NCT), Heidelberg, Germany; German Consortium of Translational Cancer Research (DKTK), Clinical Cooperation Unit Neuropathology, German Cancer Research Center (DKFZ), Heidelberg, Germany; Department of Neuropathology, Institute of Pathology, Ruprecht-Karls-University Heidelberg, Heidelberg, Germany; Clinical Cooperation Unit Neurooncology, German Cancer Consortium (DKTK), German Cancer Research Center (DKFZ), Heidelberg, Germany; Department of Neurology, University of Heidelberg Medical School and National Center for Tumor Diseases (NCT), Heidelberg, Germany; Faculty of Biosciences, Heidelberg University, Heidelberg, Germany; Clinical Cooperation Unit Translational Radiation Oncology, German Cancer Consortium (DKTK) Core Center Heidelberg, German Cancer Research Center (DKFZ), Heidelberg, Germany; Division of Molecular and Translational Radiation Oncology, Department of Radiation Oncology, Heidelberg Faculty of Medicine (MFHD) and Heidelberg University Hospital (UKHD), Heidelberg Ion-Beam Therapy Center (HIT), National Center for Tumor Diseases (NCT), Heidelberg, Germany; Clinical Cooperation Unit Translational Radiation Oncology, German Cancer Consortium (DKTK) Core Center Heidelberg, German Cancer Research Center (DKFZ), Heidelberg, Germany; Division of Molecular and Translational Radiation Oncology, Department of Radiation Oncology, Heidelberg Faculty of Medicine (MFHD) and Heidelberg University Hospital (UKHD), Heidelberg Ion-Beam Therapy Center (HIT), National Center for Tumor Diseases (NCT), Heidelberg, Germany; Clinical Cooperation Unit Neurooncology, German Cancer Consortium (DKTK), German Cancer Research Center (DKFZ), Heidelberg, Germany; Department of Neurology, University of Heidelberg Medical School and National Center for Tumor Diseases (NCT), Heidelberg, Germany; German Consortium of Translational Cancer Research (DKTK), Clinical Cooperation Unit Neuropathology, German Cancer Research Center (DKFZ), Heidelberg, Germany; Department of Neuropathology, Institute of Pathology, Ruprecht-Karls-University Heidelberg, Heidelberg, Germany; Clinical Cooperation Unit Neurooncology, German Cancer Consortium (DKTK), German Cancer Research Center (DKFZ), Heidelberg, Germany; Department of Neurology, University of Heidelberg Medical School and National Center for Tumor Diseases (NCT), Heidelberg, Germany; Department of Functional Proteome Analysis, German Cancer Research Center (DKFZ), Heidelberg, Germany; Department of Functional Proteome Analysis, German Cancer Research Center (DKFZ), Heidelberg, Germany; Clinical Cooperation Unit Neurooncology, German Cancer Consortium (DKTK), German Cancer Research Center (DKFZ), Heidelberg, Germany; Department of Neurology, University of Heidelberg Medical School and National Center for Tumor Diseases (NCT), Heidelberg, Germany; German Consortium of Translational Cancer Research (DKTK), Clinical Cooperation Unit Neuropathology, German Cancer Research Center (DKFZ), Heidelberg, Germany; Department of Neuropathology, Institute of Pathology, Ruprecht-Karls-University Heidelberg, Heidelberg, Germany; Clinical Cooperation Unit Translational Radiation Oncology, German Cancer Consortium (DKTK) Core Center Heidelberg, German Cancer Research Center (DKFZ), Heidelberg, Germany; Division of Molecular and Translational Radiation Oncology, Department of Radiation Oncology, Heidelberg Faculty of Medicine (MFHD) and Heidelberg University Hospital (UKHD), Heidelberg Ion-Beam Therapy Center (HIT), National Center for Tumor Diseases (NCT), Heidelberg, Germany; Clinical Cooperation Unit Neurooncology, German Cancer Consortium (DKTK), German Cancer Research Center (DKFZ), Heidelberg, Germany; Department of Neurology, University of Heidelberg Medical School and National Center for Tumor Diseases (NCT), Heidelberg, Germany; Clinical Cooperation Unit Neurooncology, German Cancer Consortium (DKTK), German Cancer Research Center (DKFZ), Heidelberg, Germany; Department of Neurology, University of Heidelberg Medical School and National Center for Tumor Diseases (NCT), Heidelberg, Germany

**Keywords:** brain tumor, glioblastoma, immune therapy, precision medicine, targeted therapy

## Abstract

**Background:**

Targeted immunotherapies are of growing interest in the treatment of various cancers. B7 homolog 3 protein (B7-H3), a member of the co-stimulatory/-inhibitory B7-family, exerts immunosuppressive and pro-tumorigenic functions in various cancer types and is under evaluation in ongoing clinical trials. Unfortunately, interaction partner(s) remain unknown which restricts the druggability.

**Methods:**

Aiming to identify potential binding partner(s) of B7-H3, a yeast two-hybrid and a mass spectrometry screen were performed. Potential candidates were evaluated by bimolecular fluorescence complementation (BiFC) assay, co-immunoprecipitation (co-IP), and functionally in a ^3^H-thymidine proliferation assay of Jurkat cells, a T-cell lineage cell line. Prognostic value of angio-associated migratory cell protein (AAMP) and B7-H3 expression was evaluated in isocitrate dehydrogenase 1 wildtype (IDH1wt) glioblastoma (GBM) patients from The Cancer Genome Atlas (TCGA)-GBM cohort.

**Results:**

Of the screening candidates, CD164, AAMP, PTPRA, and SLAMF7 could be substantiated via BiFC. AAMP binding could be further confirmed *via* co-IP and on a functional level. AAMP was ubiquitously expressed in glioma cells, immune cells, and glioma tissue, but did not correlate with glioma grade. Finally, an interaction between AAMP and B7-H3 could be observed on expression level, hinting toward a combined synergistic effect.

**Conclusions:**

AAMP was identified as a novel interaction partner of B7-H3, opening new possibilities to create a targeted therapy against the pro-tumorigenic costimulatory protein B7-H3.

**Key Points**

- AAMP is a binding partner of costimulatory B7-H3.
- Immunosuppressive function of B7-H3 can be blocked by targeting AAMP.

**Importance of the Study**

B7 homolog 3 protein (B7-H3), a member of the co-stimulatory/-inhibitory B7-family, was shown to be immunosuppressive and pro-tumorigenic in various cancers. Although evaluated in ongoing clinical trials, a validated binding partner in immune cells has not yet been identified. Using different screening assays, we could identify several probable interaction partners of B7-H3. For one candidate, angio-associated migratory cell protein, we validated this interaction on the functional level in immune cells and could demonstrate a combined synergistic effect on the expression level, offering new possibilities to create a targeted therapy against the pro-tumorigenic costimulatory protein B7-H3.

Targeted immuno-therapies are of growing interest in the treatment of various cancers. In particular, members of the co-stimulatory/-inhibitory B7-family have come into the focus of research within the last two decades. Immune checkpoint inhibitors have improved the treatment options and prognosis of advanced non-small cell lung cancer, melanoma, and renal cell carcinoma.^1^ Although checkpoint inhibitors demonstrated clinical benefit in some cancers, neoplasms such as glioblastoma (GBM) showed diverse responses, with discouraging results in newly diagnosed GBM^2^ and beneficial results in recurrent GBM,^3^ underlining the need for more individualized treatment options.

In this regard, B7 homolog 3 protein (B7-H3), a member of the co-stimulatory/-inhibitory B7-family, is under evaluation in ongoing clinical trials against various cancer types. Taking advantage of the upregulated expression of B7-H3 in many cancer types,^4^ antibody-dependent cell-mediated cytotoxicity (trial NCT01391143) and antibody-drug conjugates against B7-H3 (NCT01099644, NCT01502917, and NCT00089245) are under investigation.^5^ Moreover, as B7-H3 has been shown to mediate tumor supporting functions such as invasiveness, metastasis, enhanced therapy resistance, and suppression of the antitumor immune function, other trials (NCT02381314 and NCT02475213) focus on blocking B7-H3 function.^4,6–15^ Finally, B7-H3 was identified as an interesting target for CAR T-cell therapies in neuroblastoma and GBM.^16,17^

Although the importance of B7-H3 in the context of cancer has been well described, the receptor(s) to which B7-H3 binds have still not been comprehensively investigated. This may also be a consequence of B7-H3’s two-headed role in immunology as B7-H3 exerts immune-stimulating or immune-suppressing properties depending on the tumor type.^18^ In 2008, *Hashiguchi* et al. described TLT-2, triggering receptor expressed on myeloid cells (TREM)-like transcript 2 as the first potential receptor of B7-H3.^19,20^ However, the interaction between B7-H3 and TLT-2 which was not confirmed by other authors cannot explain the mostly published pro-tumorigenic function of B7-H3 as the authors describe an immune-stimulating role of the B7-H3/TLT-2 interaction.^21^

Recently, IL20-RA was claimed to be the counterreceptor of B7-H3, which is still mostly described as an orphan immune checkpoint member. Therefore, a new platform for extracellular interactome discovery was used but this interaction was not confirmed with further experiments and beyond that, a functional proof of the interaction is missing.^22^

Hence, our study aimed to identify novel interaction partners of B7-H3. Therefore, two screening assays were performed. First, a yeast two-hybrid (Y2H) screen was carried out offering the possibility to detect interaction partners beyond the B7-H3 function in the immune system. Next, mass spectrometry was employed for the detection of membrane-bound phospho-proteins of natural killer (NK) cells co-incubated with B7-H3-expressing or B7-H3-knockdown GBM cells. The latter was performed after observing that B7-H3-expressing GBM cells can suppress NK cells.^12^ Therefore, we aimed to identify proteins with immunoreceptor tyrosine-based inhibitory motif which we postulated to transduce the inhibitory signal of B7-H3 to the NK cells.

The screened candidates were further checked by bimolecular fluorescence complementation (BiFC). Here, CD164, angio-associated migratory cell protein (AAMP), PTPRA, and SLAMF7 were confirmed as potential candidates. Finally, AAMP was substantiated as an interaction partner of B7-H3 by pull-down assay and also by proving interaction on the functional level.

AAMP was described to be involved in angiogenesis and migration of endothelial cells, including cancer cells.^23,24^ Forming a WD40 domain and containing immunoglobulin-like domains, AAMP is fitted to interact with a wide plethora of different proteins. Thus, AAMP was until now published to interact with RhoA pathways,^25^ thromboxane A2 receptors,^25–27^ and take part in autophagy^28^ among others.

## Materials and Methods

### Ethics Statement

All work presented was performed with the approval (application number S310/2019) of the ethics board of the medical faculty of the University of Heidelberg, Germany.

### Cells and Cell Culture

All glioma cell lines (U87MG, T98G, LN18, LN319, U138MG, A172, U231, LN428, LN308, D247, U373, LN229, and Hs683) were maintained in Dulbecco’s modified Eagle’s medium (DMEM) media (Sigma-Aldrich) supplemented with 10% fetal bovine serum (FBS) (Sigma-Aldrich) and penicillin (100 IU/ml)/streptomycin (100 mg/ml) (P/S) (Sigma-Aldrich). LN-229 and Hs683 cell lines were kindly provided by Dr N. de Tribolet (Department of Neurosurgery, University Hospital, Lausanne, Switzerland) and Dr Björn Tews (DKFZ, Heidelberg, Germany), respectively. The lentiviral knockdown of 4Ig-B7-H3 and control knockdown LN229 cell line^12^ were cultured under selective pressure in 3 µg/ml puromycin (Applichem). Human NK cell lines, NK92Cl and NKL, were kindly provided by the laboratory of Prof. Dr Adelheid Cerwenka (Mannheim, Germany). The NK92Cl cell line was maintained in MEM Alpha (1X) (Gibco) supplemented with 12.5% FBS, 12.5% horse serum (Gibco), 2 mM l-glutamine (Gibco), penicillin (100 UI/ml)/streptomycin (100 mg/ml), and 0.05 mM β-mercaptoethanol (Sigma-Aldrich). The NKL cell line was maintained in RPMI-1640 (PAN Biotech), 10% FBS, P/S, and 100 U/ml human recombinant IL-2. The HEK293 cell line was cultivated in DMEM, 10% FBS, and P/S. All cell lines were kept under standard conditions at 37°C and 5% CO_2_.

### Primary Cell Culture

Human peripheral blood mononuclear cells (PBMCs) were maintained in RPMI-1640 supplemented with 10% human serum AB (Sigma-Aldrich), P/S, and 2 mM l-glutamine under standard conditions at 37°C and 5% CO_2_. Cell lines were authenticated using Multiplex Cell Authentication by Multiplexion (Heidelberg, Germany) as described.^29^ The absence of mycoplasma infection is screened for regularly.^29^

Glioma-initiating cells (GICs) cultures were established from freshly dissected tumor tissue. Tumor and neurosphere cultures were cultured as described. Cells were seeded in a neural sphere cell medium containing DMEM F12 enriched with B27 supplement, basic fibroblast growth factor (20 ng/mL) and epidermal growth factor (20 ng/ml).^30^

### Isolation of PBMCs and T-cells

PBMCs were isolated from buffy coats (healthy donors) by density-gradient centrifugation using Ficoll (GE-Healthcare) and T cells were isolated using the MagniSortTM Human T cell Enrichment Kit according to manufacturer’s instructions (eBioscience, order no. 8804-6810). CD4^+^ and CD8^+^ T cells were separated with the CD4 T-cell isolation kit, human (order no. 130-096-533), or by CD4 MicroBeads, human (order no. 130-045-101), on isolated T cells using magnetic-activated cell sorting (MACS) according to manufacturer’s instructions (Miltenyi Biotec).^31^ Purity was checked by flow cytometry using phycoerythrin (PE)-conjugated mouse anti-human CD3 (HIT3a, Biolegend), mouse antihuman PerCP-Cy5.5-conjugated CD8a (RPA-T8, Thermo Fisher Scientific), and mouse antihuman eFluor 450-conjugated CD4 (RPA-T4, Thermo Fisher Scientific).

### Screening Assays

#### Split Ubiquitin Y2H screen.

—Gateway compatible vectors for Split-Ubiquitin (pMet, pNuI, pCKZ, and pCup-CGK) were a kind gift by Laurent Deslandes and Imre E. Somssich (Max Planck Institute for Plant Breeding Research, Cologne, Germany) (detailed in Supplemental Methods).

#### Phospho-tyrosine screen of NK cells.

—33 × 10^6^ LN229 shB7-H3 and LN229 control cells were plated in 6 well plates for 6 h. Afterward, they were co-incubated with 10^6^ freshly isolated NK cells for 45 min. NK-cells were carefully mobilized with 5 ml of ice-cold phosphate buffered saline (PBS) and pelleted (500 g, 5 min, 4°C). After resuspension in freeze and thaw buffer (PBS, 1 mM MgCl_2_, 2 mM Na-orthovanadate, Phosphatase-Inhibitor cocktail 1 and 3 [Sigma-Aldrich]) cells were treated with 2 freezes (liquid-nitrogen) and thaw cycles. Afterward, cells were pelleted (13.000 rpm, 4°C, 1 h) to separate the cytoplasmic lysis fraction (supernatant) from the membrane fraction. The latter one was lysed for 30 min in digitonin lysis buffer (150 mM NaCl, 1 mM MgCl_2_, 10 mMTris-HCl [pH8], 1% digitonin, 2 mM Na-orthovanadate, Phosphatase-Inhibitor cocktail 1 and 3 [Sigma-Aldrich]) and afterward pelleted (13.000 rpm, 4°C, 10 min). Supernatant was kept for analysis of phosphoproteome by mass spectrometry (detailed in Supplemental Methods).

### Immunohistochemistry

Formalin-fixed paraffin-embedded tissues of human astrocytoma, oligodendroglioma, and GBM were provided by the Neuropathology Department of University Hospital Heidelberg, Germany. The diagnoses for the current study had been based on WHO classification 2016. Three-micrometer cut sections were processed with Ventana BenchMark XT immunostainer (Ventana Medical Systems). The staining procedure includes the incubation of Ventana Cell Conditioner-treated samples with antihuman AAMP (rabbit anti-AAMP; 1:200, Abcam) primary antibody at 37°C for 32 min, and secondary antibody (P0446; DAKO) at radiotherapy (RT) for 32 min. Further staining procedure is as described before.^12^

### BiFC Assay

Using the gateway cloning system (Invitrogen), B7-H3 and the extracellular fragments of the screened binding partners bought as codon-optimized GeneArt Strings DNA Fragments from Invitrogen (Supplemental Methods) were cloned into Myc-LC151 and Ha-LN151 containing each a complementary fragment of a fluorescent reporter protein. HEK293 cells were co-transfected with Myc-B7-H3 and Ha-AAMP, vice versa. Upon close proximity of the two proteins tested, the both hemi-fluorescent reporters emitted fluorescence and could be observed under a Leica DM IRB microscope.

### Lentivirus Production

HEK293T cells were co-transfected with shERWOOD UltramiR targeting vectors to generate a non-targeting control, or three different human AAMP-targeting lentiviral particles (ULTRA-3219597, -3219598, -3219599; Transomic). The shERWOOD UltramiR lentiviral vectors were diluted to 0.2 μg/μl plasmid mix containing 0.5 μg/μl psPAX2, 0.5 μg/μl pMD2.G, and 0.3 μg/μl esiRNA human dgcr8 (esiRNA1; Sigma-Aldrich). The plasmid mix was added to 850 μl RPMI-1640 and 50.4 FuGENE HD (Promega), and incubated for 15 min at RT before adding dropwise onto HEK293T cells seeded the day before. The viral particles were collected 48 h later and centrifuged for 10 min at 800*g*.

#### Lentiviral knockdown of AAMP.

—Jurkat cells were incubated in 500 μl lentivirus and 50 μg/μl Protransduzin-A (Immundiagnostik) for 5 min at RT. The Jurkat cells were transferred to fresh media after 4 h, and were selected with 1 μg/μl puromycin (AppliChem). Transduction efficiency was determined by FACSCanto II. The knockdown was verified by immunoblotting.

### Western Blot

Total cell lysates were generated in 50 mM Tris-HCl (pH 8.0), 150 mM NaCl, 1% Nonidet P-40 (Genaxxon Bioscience), 10 mM EDTA, 100 mM PMSF, 200 mM DTT (Carl Roth), cOmplete (Roche), and phosphatase inhibitor cocktail 2 and 3 (1:100, Roche). Protein concentrations were measured via Bio-Rad protein assay (Bio-Rad). SDS-PAGE separated samples were transferred to nitrocellulose membrane. After blocking the membranes in 5% milk powder, membranes were incubated with human targeting primary antibodies goat anti-B7-H3 (CD276) (1:5000, R&D Systems), rabbit anti-AAMP (1:5000, Abcam), goat anti-GAPDH (1:1000, Linaris), mouse anti-α-Tubulin (1:500, Sigma-Aldrich), rabbit anti-β-Actin (1:1000, Cell Signaling). Secondary antibodies were horseradish peroxide-conjugated donkey anti-goat immunoglobulin G (IgG) (1:5000, Santa Cruz Biotechnology), sheep anti-mouse IgG (1:5000, Sigma-Aldrich), donkey anti-rabbit IgG (1:5000, Sigma-Aldrich), and mouse anti-rabbit light chain (1:10.000, Merck). Chemiluminescence development was performed using enhanced chemiluminescence (ECL) or ECL prime (Amersham) and visualized by developing Super RX-N films or ChemiDOC MP Imaging System (BioRad).

### Co-immunoprecipitation

To show the interaction between B7-H3 and AAMP, two different co-immunoprecipitation (co-IP) assays were performed.

1. Halo-tagged-B7-H3 overexpressing HEK293 cells were cultivated overnight. After washing the HEK293 cells with cold PBS, the cells were lysed in NP40 lysis buffer. Cells were incubated on ice for 5 min and centrifuged at 600 g at 4°C for 10 min. The supernatant containing Halo-tagged B7-H3 was incubated with Magne HaloTag beads overnight at 4°C. In parallel, Jurkat cells were cultured overnight. The next day Jurkat cells and the Magne HaloTag beads bound to Halo-tagged-B7-H3 were incubated together. Next, the samples were lysed in 1 ml 100 mM octyl glucoside and the beads were separated from the supernatant with a magnetic sorter (Miltenyi Biotec). After washing, the co-precipitated proteins were released from the beads by overnight incubation in purification buffer containing tobacco etch virus (TEV) protease at 4°C. Supernatant was collected for further western blot analysis.
2. NKL cells were lysed in NP40 lysis buffer. The supernatant was incubated with recombinant B7-H3 for 1 h. Subsequently, anti-goat IgG or anti-human B7-H3 was added into the supernatant to incubate for 30 min at 4°C. Following the addition of Dynabeads Protein G, the samples were left to incubate overnight. The next day, beads were washed and separated from the supernatant by adding 1X Laemlli buffer diluted in lysis buffer and boiling at 95°C for 10 min. The co-precipitated samples were assessed by western blotting.

### ^*3*^H-Thymidine Proliferation Assay

To assess the proliferation of Jurkat cells ^3^H-Thymidine incorporation was measured. Fifteen thousand cells/well were dissociated and plated in quadruplicates in 96-well plates in 200 μl volumes of medium. B7-H3 (Catalog number 11188-H08H-B; BIOZOL, Eching, Germany) or buffer control was added as indicated. After 72 h, cells were pulsed for 24 h with [methyl-3H] thymidine (0.5 lCi), harvested (Tomtec, Hamden, CT), and incorporated radioactivity was determined in a liquid scintillation counter (Wallac, Turku, Finland).^32^

### Statistics

The proliferative effect of B7-H3 on AAMP was analyzed in 4 independent experiments with 4 technical replicates each. To facilitate comparison between individual experiments, units were log-transformed and the mean values of the respective B7-H3 control groups (knockdown control and AAMP knockdowns) were subtracted from the individual values in the B7-H3-treated groups. Two-way ANOVA on single values was performed to test the influence of B7-H3 treatment (no B7-H3, 20 ug/ml B7-H3, 30 ug/ml B7-H3, and 40 ug/ml B7-H3) on AAMP knockdown (shAAMP 1 and 2) and control. One-way ANOVA on single values was performed to test the influence of B7-H3 treatment (no B7-H3 and 20 ug/ml B7-H3) on AAMP knockdown and control. Significance was indicated at *P* < .05. Dunnett's test was used for pairwise post hoc comparisons of factor levels. Data are presented as individual values, color-coded dependent on the biological replicate, and mean ± SEM). Statistical tests were performed using GraphPad Prism 8.3.0 (GraphPad Software, San Diego, CA)

### Molecular Data and In silico Analysis

TGCA-GBM RNASeq and whole exome sequencing expression data were retrieved from the GDC data portal and RNASeq data was vst normalized (DESeq2^33^). Only isocitrate dehydrogenase 1 wildtype (IDH1wt) samples were included in subsequent analyses.

Statistical analyses were performed with R version 4.1.2.^34^ Survival data were analyzed with parametric models with the survival package,^35^ assuming a Weibull distribution. Optimal cutoffs of group definition were selected based on minimal *P*-values (dataAnalysisMisc package^36^). Associations with gene expressions were computed with linear models, *P*-value was multiplicity adjusted. The umap package^37^ was used to obtain low dimensional representations from gene set expression data. Testing for significant interaction values for varying cutoffs of B7-H3 and AAMP umap representations was performed with parametric survival models (Weibull distribution), including only interactions. Pathway enrichment analyses were performed with the enrichR package.^38^

Preprocessed single cell 10X GBM IDH1wt data was downloaded from GEO (GSE131928/GSM3828673), and analyzed/visualized with Seurat^39^ standard workflow (NormalizeData, FindVariableFeatures, ScaleData, RunPCA, FindNeighbors [dims = 1:10], FindClusters [resolution 0.8], RunUMAP [dims = 1:10]). Single cell GBM data and t-distributed stochastic neighbor embedding (tSNE) representation with cell type assignment from 4 tumors was retrieved from gbmseq.org.^40^ Interactive low-dimensionality data from https://www.brainimmuneatlas.org/^41^ was analyzed and representative figures were obtained from this atlas from multiple GBM single cell datasets (*n* = 7 newly diagnosis GMB, *n* = 4 recurrent GBM).

## Results

### Identification of Glioma-Derived B7-H3 Binding Partners on NK Cells

**Figure 1.**
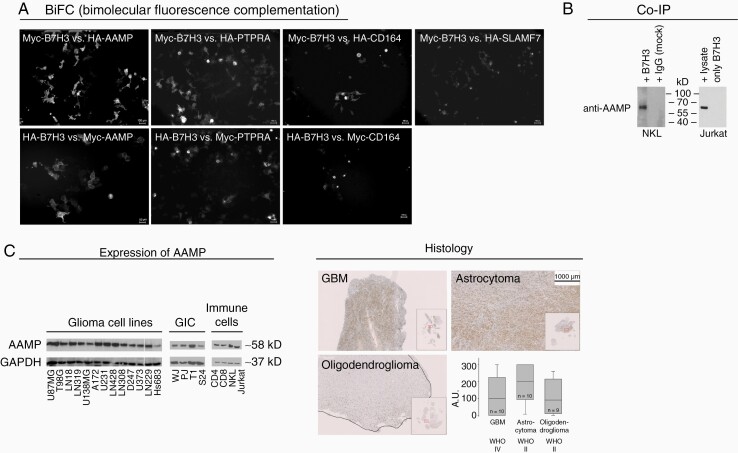
Identification of new binding partners of B7-H3. (**A**) Potential binding partners, identified by mass spectrometry and yeast two-hybrid screen were evaluated by BiFC. AAMP, CD164, and PTPRA showed a fluorescence signal regardless of whether they were expressed in the Myc- or HA-vectors expressing a hemi-fluorescence protein when co-expressed with Myc- or HA-vector cloned B7-H3 expressing the other half of the fluorescence protein. SLAMF7 showed a signal only when cloned into the HA-vector when co-expressed with Myc-B7-H3. (**B**) Co-immunoprecipitation using recombinant B7-H3 which was pulled down with anti-B7-H3 antibody (NKL) or via a TEV-tag (Jurkat) cells confirms AAMP as a binding partner of B7-H3 in untransduced natural killer (NKL) and T-cell (Jurkat) lines. (**C**) AAMP is ubiquitously expressed in different glioma cell lines, glioma initiating cultures (GIC), freshly isolated CD4 and CD8 T cells as well as NKL and Jurkat immune cell lines. (**D**) AAMP expressed in tumor tissue of glioblastoma, astrocytoma, and oligodendroglioma does not correlate with tumor grade (magnification: 1:10).

To identify potential extracellular binding partners of tumor-derived B7-H3 on NK cells, Y2H (Supplemental Table 1) and a mass spectrometry phosphorylation screen (Supplemental Table 2) were performed. In the mass spectrometry phosphorylation screen, potential binding partners of B7-H3 were identified by analyzing tyrosine-phosphorylation sites of NK cells, which were confronted with LN229 GBM cells silenced for B7-H3 or with control-LN229 cells presenting B7-H3. The results of the two screens were narrowed down to 17 genes following a literature review. As we were interested in extracellular binding partners of B7-H3, genes shown to have an extracellular domain or to be secreted into the extracellular space were considered further. We were preferentially interested in candidates proposed to exert an immunologic function. Codon-optimized DNA strings consisting of only the transmembrane and extracellular domain of the selected 17 genes (Supplemental Figure 1) were generated to be analyzed for their interaction with B7-H3. The candidate genes were cloned utilizing the Gateway system. To confirm their interaction with B7-H3, BiFC assays were performed, which revealed four candidate genes: CD164, AAMP, PTPRA, and SLAMF7 as potential interactors of B7-H3 (Figure 1A). As the BiFC assay was performed by overexpressing recombinant proteins, the interaction with endogenously expressed binding partners was further assessed by co-IP. Since the AAMP candidate has been described to take part in migration and immune response, the co-IP experiments were designed to investigate the interaction between B7-H3 and endogenous AAMP. To this end, recombinant B7-H3 was used as bait for AAMP from Jurkat cells, which express AAMP (Figure 1C). The co-IP experiments verified an interaction between B7-H3 and AAMP, which was also validated utilizing the human NK-cell line, NKL (Figure 1B). Since B7-H3 has a role in immune suppression, the expression of AAMP was further examined in glioma cell lines, GICs, immune cell lines, and primary immune cells, which were all positive for this binding partner (Figure 1C). Finally, as B7-H3 expression in glioma is grade-dependent,^12^ it was tested whether AAMP is also expressed depending on glioma grade. Primary glioma tissues were assessed by immunohistochemistry. Although AAMP was clearly expressed by GBM, oligodendroglioma, and grade II astrocytoma, unlike B7-H3, it did not show a grade-dependent expression (Figure 1D). To get a clearer insight about tumor- and immune-cell expression of AAMP and B7-H3, more detailed in silico analyses were performed (Supplemental Figures 2 and 3). A single cell 10X GBM RNA-sequencing dataset of 28 GBMs^42^ allowed us to visualize the distribution of AAMP and B7-H3 expression in different GBM subsets such as mesenchymal-, astrocytic-like, oligodendrocyte progenitor-like- and neural progenitor-like cells. Moreover, we could demonstrate co-expression of AAMP and B7-H3 in immune cells such as CD68-positive macrophages as well as CD4 and CD8A positive lymphocytes. In general, AAMP-expression was more ubiquitous while B7-H3 expression was more distinct in the different subsets as detailed in Supplemental Figure 2. Interestingly, the highest expression of B7-H3 and AAMP was found in the vimentin-positive subset of mesenchymal GBM cells.

Single-cell GBM data and tSNE representation with cell type assignment from four GBMs retrieved from gbmseq.org^40^ as well as interactive low-dimensionality data from the brain immune atlas^41^ of 7 newly diagnosed GBM and 4 recurrent GBM could further substantiate our expression data (Supplemental Figure 3). The first analysis demonstrated expression of AAMP and B7-H3 in neoplastic cells as well as in myeloid cells (Supplemental Figure 3A and B). The later analysis showed expression of both AAMP and B7-H3 in tumor-associated macrophages while lymphocytes expressed mainly AAMP but not B7-H3 (Supplemental Figure 3C).

### AAMP Is a Functional Interaction Partner of B7-H3

**Figure 2.**
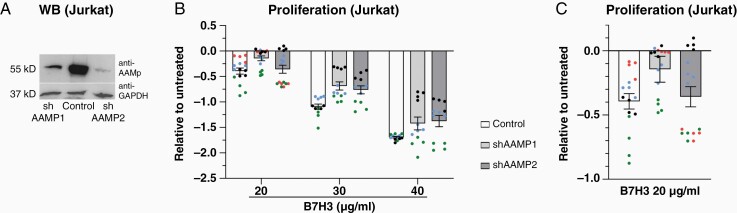
AAMP conveys the anti-proliferative effect of B7-H3 to Jurkat cells. (**A**) Knockdown control of shAAMP1 and 2 (52 kD) in Jurkat cells compared with Control confirmed by western blot. (**B**) Effect of B7-H3 treatment (20, 30, and 40 µg/ml) on AAMP knockdown (shAAMP1 and 2) and control Jurkat cells on proliferation. Proliferation was measured by the incorporation of ^3^H thymidine into the DNA. Biological replicates are indicated by different colors. *N* = 16 replicates for 20 µg/ml B7-H3 and *N* = 12 for 30 and 40 ug/ml B7-H3. (**C**) Effect of B7-H3 treatment (20 µg/ml) on the proliferation of AAMP knockdown in Jurkat cells. Proliferation was measured by the incorporation of ^3^H thymidine into the DNA and shows the specific reduction of the inhibitory B7-H3 function in shAAMP-Jurkat cells. Biological replicates are indicated by different colors. *N* = 4 replicates. Data are individual values and means ± SEM.

To test whether B7-H3 interacts on a functional level with AAMP, two AAMP knockdown cell lines (AAMP 1 and AAMP 2) were successfully created in an immortalized cell line of human T lymphocyte (Jurkat) cells (Figure 2A). There was a significant effect of the B7-H3 regime (20, 30, and 40 μg/ml) on proliferation of Jurkat lines, showing that higher concentrations of B7-H3 significantly inhibited proliferation of both Jurkat control and AAMP-knockdown cells (Figure 2B) (*P* < .05). At the lowest dosage of B7-H3 (20 µg/ml), proliferation of the Jurkat control cells was significantly stronger inhibited compared to the AAMP knockdown Jurkat cell lines (Figure 2C) (*P* < .05). Taken together, these results suggest that knockdown of AAMP in Jurkat cells inhibits the antiproliferative effect of B7-H3. This demonstrates that B7-H3 exerts its anti-proliferative effect in part by interaction with AAMP.

### Prognostic value of AAMP and B7-H3 in GBM

**Figure 3.**
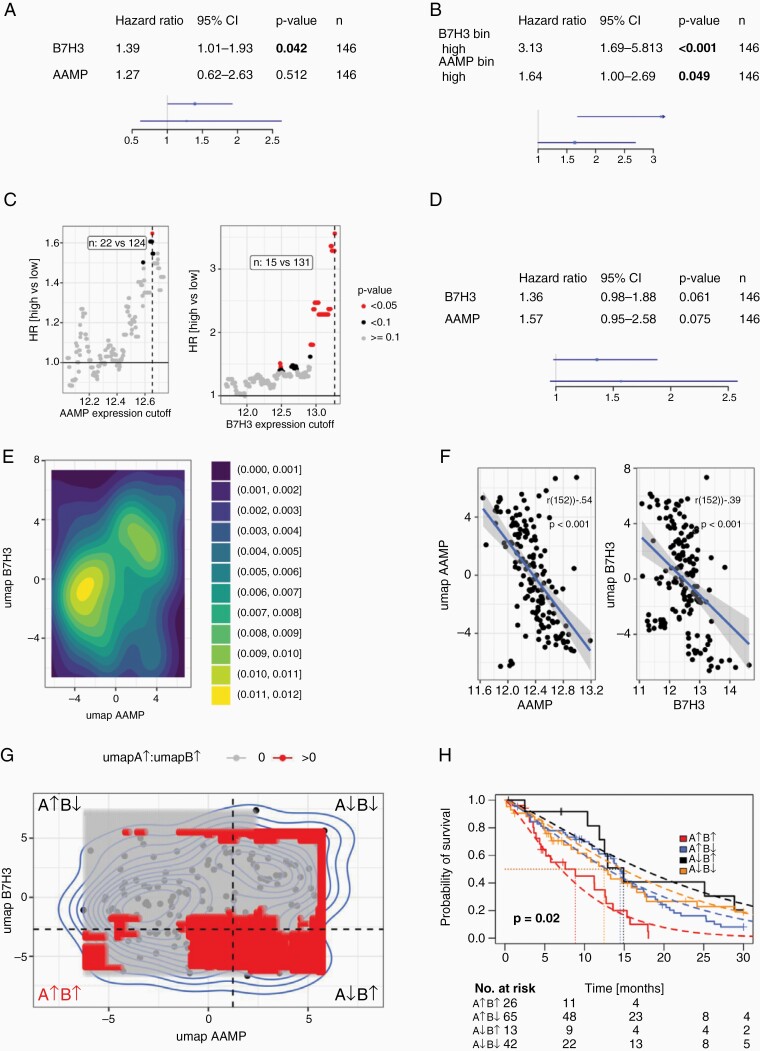
Prognostic value of AAMP and B7-H3 expression in IDH1wt GBM (TCGA-GBM). Univariate survival analysis with continuous expression level covariate (**A**) and binarized groups (**B**). (**C**) shows the model performance depending on the selected gene expression cutoff, dashed lines correspond to cutoffs shown in **B + C**. (**D**) Multivariate survival analysis. (**E**) Density plot of 1d-umap data for genes associated with AAMP (*n* = 110) and B7-H3 (*n* = 434) expression (*P*_Bonf_ < .001, upper and lower 1% quantile of coefficients). (**F**) Association of AAMP (**A**) and B7-H3 (**B**) gene expression with umap values, Pearson correlations. **(G)** Testing for significant interactions between umap value defined groups, four quadrants are labeled with corresponding AAMP (**A**) and B7-H3 (**B**) gene expression. Non-transparent (red) shows areas with significant effects for A↑B↑. Dashed lined indicate lowest observed p-value, corresponding groups are shown in (**H**) (parametric survival models, Weibull distribution).

The prognostic value of AAMP and B7-H3 expression was evaluated in IDH1wt GBM patients from The Cancer Genome Atlas (TCGA)-GBM cohort (Figure 3). Univariate survival analysis using gene expression as continuous covariates showed a negative prognostic value for B7-H3 but not for AAMP (Figure 3A). Separation into two groups based on a varying cutoff also revealed a prognostic value for AAMP (Figure 3B and C). Especially high expression (cutoff values > 12.5) lead to significant separation. Multivariate analysis of both genes showed a trend (Figure 3D), hinting toward independent latent factors. To further examine this finding, we evaluated genes showing high association with AAMP and B7-H3 expression, using strict filtering criteria: Bonferroni *P*-value adjustment, cutoff 0.001, only genes with highest and lowest 1% of observed effects/ coefficients. 1d UMAP values were computed from selected genes (AAMP: *n* = 110 genes; B7-H3: *n* = 434), hinting toward two distinct populations (Figure 3E). Single umap values showed high correlation with their respective gene expression values (Figure 3F). To test for a potential interaction effect, four groups were formed for varying cutoffs (Figure 3G), and if a significant interaction was observed (*P* < .05), the respective cutoff was marked (red area in Figure 3G). The lowest *P*-value is marked with dashed lines and the corresponding Kaplan-Meier survival plot is depicted in Figure 3H. Here, a poor prognosis could be observed for patients with high AAMP and B7-H3 expression (red curve), whereas high B7-H3 expression and low AAMP showed a better prognosis. This separation was not observed in the B7-H3 low group. Therefore, an interaction between AAMP and B7-H3 can be observed on the expression level, hinting toward a combined synergistic effect.

## Discussion

We present a new interaction between human B7-H3 and the AAMP. The first evidence of the interaction was given by a Y2H screen. The interaction could be confirmed by BiFC, pull-down assays, and finally on a functional level, showing that the immunosuppressive function of B7-H3 on the proliferation of Jurkat cells is in part conveyed by AAMP. Hence, knockdown of AAMP reduced specifically the anti-proliferative effect of B7-H3 in Jurkat cells. Our results indicate that AAMP is not the only interaction partner of B7-H3 in Jurkat cells, as the anti-proliferative effect of B7-H3 was not completely neutralized. Increasing concentrations of recombinant B7-H3 were still capable to suppress the proliferation of shAAMP-Jurkat cells (Figure 2B). Furthermore, a difference between the proliferation of shAAMP- and shControl-Jurkat cells could only be recorded at the lowest concentration of recombinant B7-H3 treatment arguing for additional interaction partners of B7-H3 that compensate for the immunosuppressive function of AAMP with mounting doses of B7-H3. This might also be an indication of a cooperating action of B7-H3 and AAMP on a common receptor which can be substituted by higher doses of B7-H3.

To substantiate the finding that AAMP and B7-H3 are interaction partners, the prognostic value of AAMP and B7-H3 expression was evaluated in IDH1wt GBM patients from the TCGA-GBM cohort (Figure 3). Multivariate analysis of expression of both genes showed a trend (Figure 3D), hinting toward independent latent factors. Of note, a poor prognosis could only be observed for patients with high AAMP and B7-H3 expression, supporting the interaction between AAMP and B7-H3 on expression level and toward a combined synergistic effect.

Unfortunately, other potential binding partners such as SLAMF7, PTPRA, and CD163 which were identified in the screening assay, and partly verified by BiFC could not be further substantiated by pull-down assays. This might be due to a weaker or shorter interaction, technical issues such as poorly evaluated antibodies against these less known proteins, and the necessity for different culture models. Furthermore, Y2H systems are known to produce false-positive hits. In our Split-Ubiquitine system, there is a known bias toward membrane and cytosolic proteins, due to the detection system that utilizes Ubiquitinase, which is not very common in the nucleus. The interaction partners from our screen that could not be validated may be detected in Y2H due to unphysiological high expression in this system, incorrect folding of the proteins due to missing chaperons, or incorrect post-translational processing of the proteins. These restraints might also explain, why other published interaction partners such as IL-20-RA could not be identified with our screens.

Interestingly, tumors are capable of specifically modulate the glycosylation pattern of B7-H3 as shown for oral cancer which also has an impact on the interaction with their receptors.^43^ In this regard, it is entirely possible, that recombinant B7-H3 produced in HEK-cells does not mimic the necessary glycosylation pattern required to successfully perform a pull-down assay in our models. Moreover, it might be necessary to perform further pull-down assays with different subsets of immune cells to confirm other potential candidates.

Interestingly, it has been shown that AAMP is upregulated in some cancers and correlated with a worse prognosis.^25,26^ Moreover, AAMP has the capacity to take part in the activation/inhibition of the innate and adapted immune system by its interaction with nucleotide-binding oligomerization domain-containing protein 2, an intracellular pattern recognition receptor having an important role in recognizing bacterial peptidoglycans and stimulating immune reactions.^44,45^ Noncanonical activation of NOD1/2 via AAMP can modulate the Nf-B-pathway, receptor-interacting serine/threonine-protein kinase 2 involved in programmed cell death, autophagy, and mitogen-activated protein kinase pathway.^44–47^ These pathways are suitable to explain the pro-tumorigenic and immunosuppressive action of B7-H3 via AAMP.

To conclude, we have identified new potential binding partners of the costimulatory B7-H3 by using different screening assays. While SLAMF7, PTPRA, and CD163 could be confirmed only by BiFC assays, further validation experiments are required. AAMP was further substantiated by pull-down assays and on the functional level. We demonstrate in detail the ubiquitous expression of AAMP in the tumor and immune compartment, proving that AAMP is a rational candidate and can explain the immunosuppressive, pro-tumorigenic function of B7-H3. AAMP is the first potential binding partner of B7-H3 that could be confirmed on the functional level and proved synergism with B7-H3 on the expression level. In view of ongoing trials attempting to target B7-H3 to overcome its immunosuppressive nature and to target various cancers due to their specific upregulation of B7-H3, our results offer an alternative possibility by blocking the interaction of B7-H3 and AAMP.

## Supplementary Material

vdac098_suppl_Supplementary_Table_S1

vdac098_suppl_Supplementary_Table_S2

vdac098_suppl_Supplementary_Figure_S2

vdac098_suppl_Supplementary_Figure_S3

vdac098_suppl_Supplementary_Data

## Notes

**Authorship Statement**: **Conceptualization**: D.L., A.A., and W.W; **methodology**: D.L., St.P., S.C., M.K., J.F., A.A., W.W., U.W., M.W., M.S., F.S., A.v.D.; **software**: D.H., M.K.,J.F.; **validation**, D.L., D.H., A.A., W.W.; **formal analysis**: D.L., M.K.,J.F.,A.A.,W.W.; **investigation**: D.L., St.P., So.P., A.B., S.C.,J.F.,M.K., U.W., L.K.; **resources**: W.W., A.A..; **data curation**: D.L., W.W., A.A., U.W., M.K.; **writing/original draft preparation**: D.L., S.C.,A.W.G.; **visualization**: D.L., L.K., S.C..; **supervision**: D.L., W.W., A.A..; **project administration**: D.L., W.W.; **funding acquisition**: W.W. All authors have read and agreed to the published version of the manuscript.
