# Supplementary figures and images for: AAMP is a binding partner of costimulatory human B7-H3
Source: Neurooncol Adv. 2022 Jun 30;4(1):vdac098. doi: 10.1093/noajnl/vdac098 (PMC9341442; doi:10.1093/noajnl/vdac098)

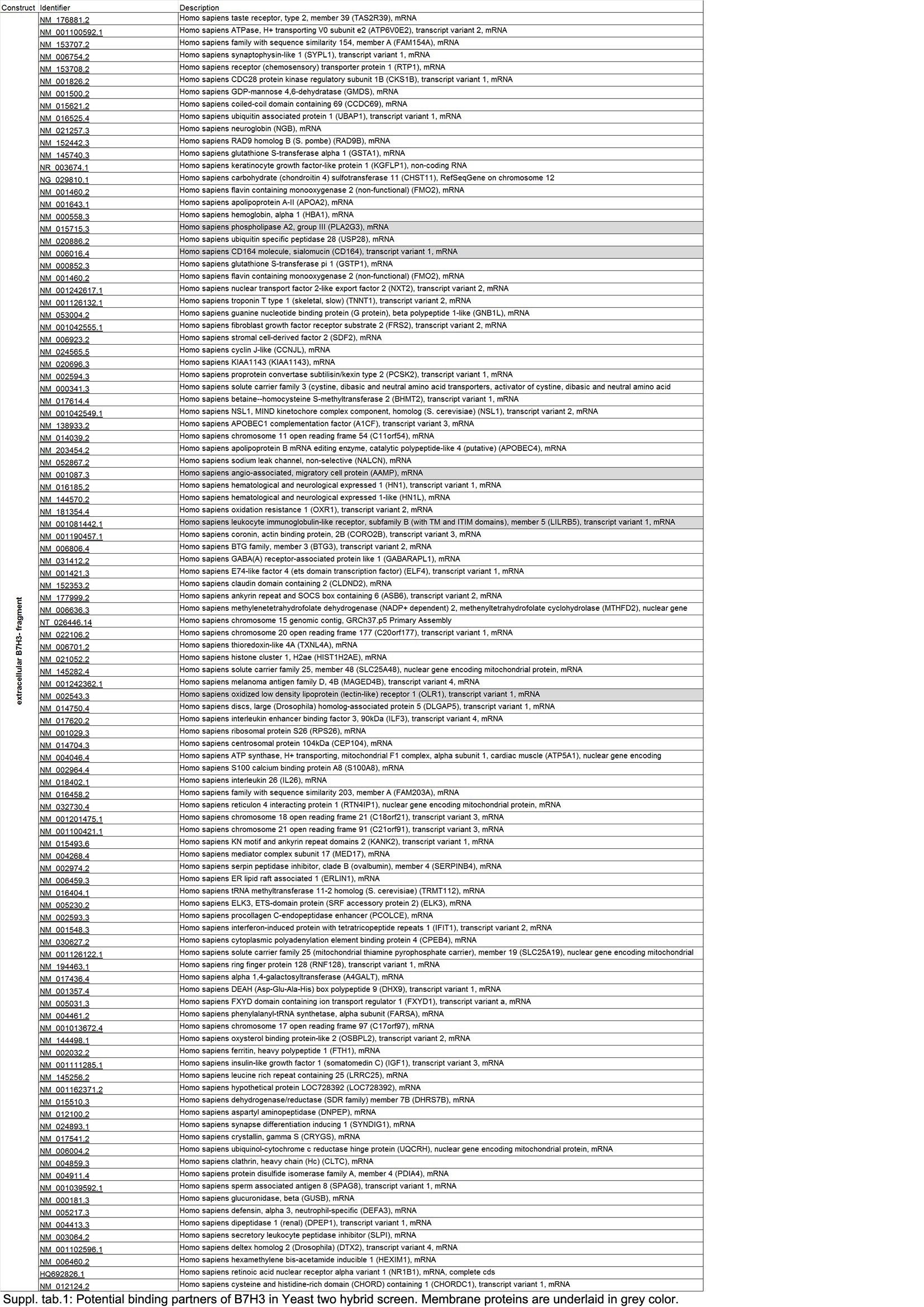

Supplement: vdac098_suppl_Supplementary_Table_S1 [file vdac098_suppl_supplementary_table_s1.jpeg]

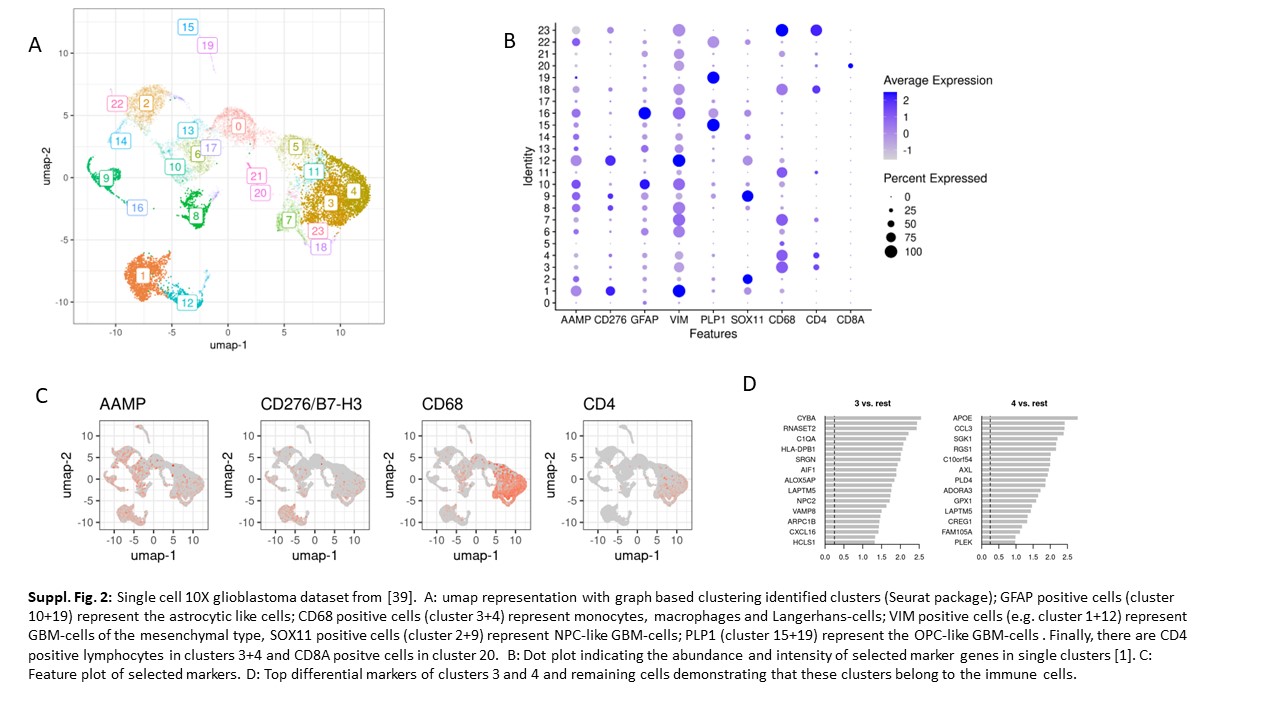

Supplement: vdac098_suppl_Supplementary_Figure_S2 [file vdac098_suppl_supplementary_figure_s2.jpeg]

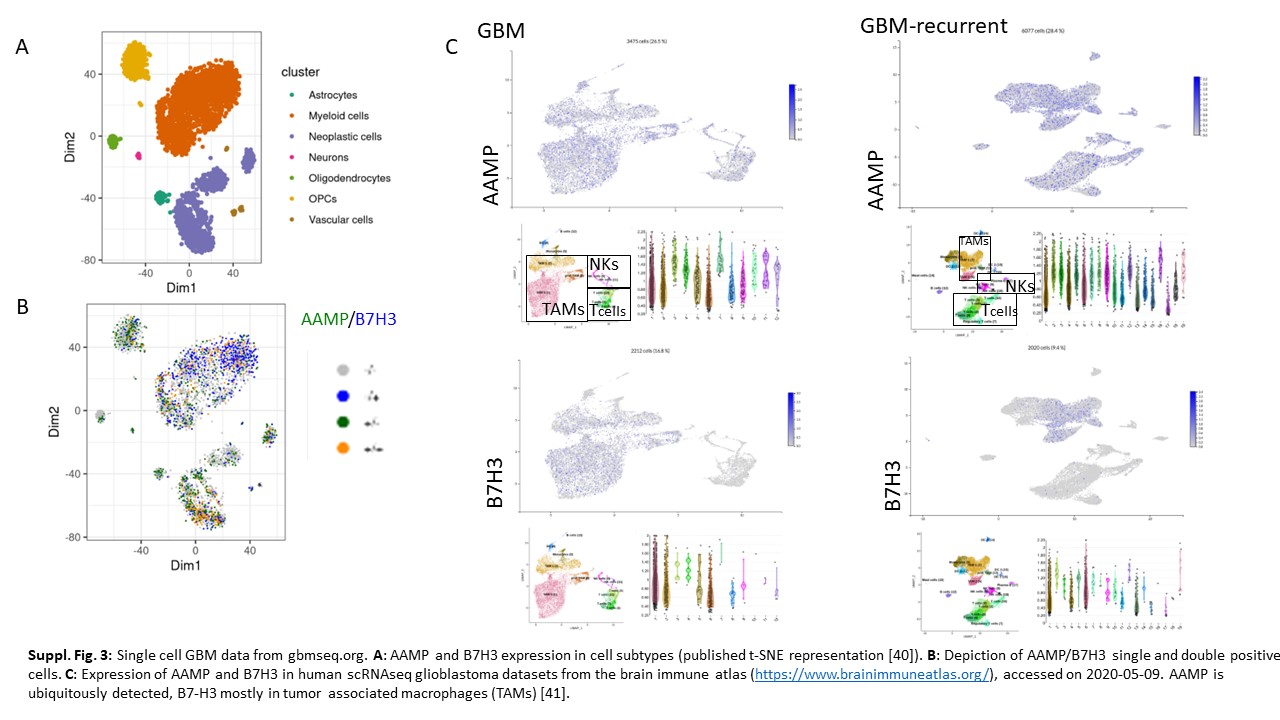

Supplement: vdac098_suppl_Supplementary_Figure_S3 [file vdac098_suppl_supplementary_figure_s3.jpeg]
