# Supplementary material for: AAMP is a binding partner of costimulatory human B7-H3
Source: Neurooncol Adv. 2022 Jun 30;4(1):vdac098. doi: 10.1093/noajnl/vdac098 (PMC9341442; doi:10.1093/noajnl/vdac098)
Supplement: vdac098_suppl_Supplementary_Data [file vdac098_suppl_supplementary_data.docx]

**Supplemental methods:**

**1. Split Ubiquitin Yeast Two-Hybrid Screen**

For all yeast experiments, we used the *Saccharomyces cerevisiae* strain JD53. For the yeast two-hybrid screen B7-H3 the CDS –STOP Codon of the long and short fragment in pDONR201 was inserted by LR-reaction (Thermo) into the bait plasmid pMet and subsequently transformed into JD53. After heat shock transformation yeast cells were streaked out on a synthetic drop-out (SD) medium for selection. Eight colonies were tested for expression and toxicity with a promoter test on SD plates with different Methionine concentrations (0, 25 and 75 µM) for promoter expression control. The best clone and Methionine concentration were chosen for cDNA library transformation. The cDNA library was a kind gift from the DKFZ Genomics and Proteomics Core Facility and includes more than 11.000 sequence-verified CDSs. The screen was performed on SD supplemented with 1 mg/mL 5-FOA and 100 mM copper sulfate. Yeast cells were grown at 30°C for 2 weeks. Positive colonies were harvested into liquid SD-media. Liquid cultures were grown at 30°C for 3 days and re-plated on SD without uracil supplemented with 100 mM copper sulfate. All colonies (116 long fragment, 125 short fragment), which grew on these plates, were used for colony PCR. For the colony PCR, part of a colony was picked into 50 μl 20 mM NaOH and lysed for 5 min. The lysate was centrifuged at 1000 rpm for 1 min and 5 μl of the supernatant was used for the PCR. The PCR consists of 1μl of each primer (P73: GTATCAATTGCATTATAATATCTTCTTGTTAGTG, P74: CTTTTCGGTTAGAGCGGATGTG), 20 μl 10x GoTaq-Polymerase master mix (Promega) and 13 µl sterile water. The PCR-protocol was 5 min at 95°C for denaturation, 30 times 95°C for 30 sec followed by 60°C 45 sec for primer binding and 72°C 150 sec elongation time.

The PCR products of all chosen positive colonies were sequenced with primer SP75 (GTGCAATATCATATAGAAGTCATCGAAATAG) at GATC Biotech AG (Konstanz, Germany). The resulting sequences were analyzed with BLAST (http://blast.ncbi.nlm.nih.gov/Blast.cgi). Out of 116 colonies for the long fragment, 110 reads included CDS and were suitable for further analysis. For the short fragment, 108 out of 125 reads included CDS and were suitable for further analysis.

**2. Phospho-tyrosine screen of NK cells**

**Phosphoproteome Analysis-Digestion and Enrichment:**

Phosphoproteome analyses were performed as previously described [31]. In brief: 100 μg protein of each lysate was used for phosphoproteome enrichment. Protein lysates were concentrated according to Wessel and Flügge [32]. Prior to trypsin digestion, the proteins were reduced and alkylated applying DTT and IAA. After digestion, samples were desalted with SPE cartridges using oligo R3 material (Life Technologies). For phosphopeptide enrichment, we used a combination of IMAC and TiO_2_ absorption techniques. First, we incubated the samples with the IMAC material in a sample/IMAC ratio of 1:10 for 1.5 h. The supernatants from the IMAC enrichment were incubated separately with TiO_2_ in a sample/TiO_2_ ratio of 1:8. Prior to LC-MS/MS analysis, samples were desalted using C18 StageTips.

**Liquid Chromatography-Mass spectrometry**

Peptide samples were separated using a nanoAcquity UPLC system (Waters). Peptide trapping was performed using a C18 precolumn (180 μm × 20 mm) with a particle size of 5 μm (Waters). Liquid chromatography separation was performed on a BEH130 C18 main-column (100 μm × 100 mm) with a particle size of 1.7 μm (Waters). The proteome samples were separated by a 1 h stepped linear gradient from 0 to 85% MeCN at a flow rate of 0.4 μl/min. The nanoUPLC system was coupled online to a nanoESI-LTQ-Orbitrap XL mass spectrometer (Thermo Scientific) in the positive ion mode at 2.4 kV. The mass spectrometer was operated in data-dependent mode to automatically measure MS1 and MS2. Data were acquired by scan cycles of one FTMS scan with a resolution of 60,000 at *m*/*z* 400 and a range from 300 to 2000 *m*/*z* in parallel with six MS/MS scans in the linear ion trap of the most abundant precursor ions.

**Data validation and classification**

LC-MS/MS raw data were processed with the Mascot search engine version 2.4 (Matrix Science) against the human SwissProt database version 2013_02 ([UniProt Consortium](https://www.uniprot.org/help/about), 539165 sequences; 191456931 residues). Cysteine carbamidomethylation was used as fixed modification; methionine oxidation, deamidation of asparagine as well as glutamine and additionally for the phosphoproteome data set serine, threonine, and tyrosine phosphorylation as variable modifications. A false discovery rate (FDR) of 0.01 for proteins, peptides and sites, a minimum peptide length of 7 amino acids, a mass tolerance of 7 ppm for precursor and 0.5 Da for fragment ions were required. Enzyme specificity was set to trypsin/p with an allowed maximum of two missed cleavages for the proteome analysis and four missed cleavages for the phosphoproteome analysis.

*DNA inserts used for Gateway Cloning (GeneArt® Strings™ DNA Fragments from Invitrogen)*

***AAMP***

*GGGGACAAGTTTGTACAAAAAAGCAGGCTCCACCATGGAAAGCGAGAGCGAGTCTGGCGCCGCTG*

*CCGATACACCTCCTCTGGAAACCCTGAGCTTCCACGGCGACGAAGAGATCATCGAGGTGGTGGAA*

*CTGGACCCCGGACCCCCCGATCCTGATGATCTGGCCCAGGAAATGGAAGATGTGGACTTCGAGGA*

*AGAAGAAGAGGAAGAGGGCAACGAAGAGGGCTGGGTGCTGGAACCTCAGGAAGGCGTCGTGGGCA*

*GCATGGAAGGCCCCGACGATAGCGAAGTGACCTTCGCCCTGCACAGCGCCAGCGTGTTCTGCGTG*

*TCCCTGGACCCCAAGACCAACACCCTGGCTGTGACAGGCGGCGAGGACGACAAGGCTTTTGTGTG*

*GCGGCTGAGCGACGGCGAGCTGCTGTTTGAATGTGCCGGCCACAAGGACAGCGTGACCTGCGCCG*

*GCTTTAGCCACGATAGCACCCTGGTGGCCACAGGCGATATGAGCGGCCTGCTGAAAGTGTGGCAG*

*GTGGACACCAAAGAGGAAGTGTGGTCCTTCGAGGCCGGCGACCTGGAATGGATGGAATGGCACCC*

*TAGAGCCCCCGTGCTGCTGGCCGGAACAGCCGATGGAAACACCTGGATGTGGAAGGTGCCCAACG*

*GCGACTGCAAGACCTTCCAGGGCCCCAACTGTCCTGCCACCTGTGGCAGAGTGCTGCCCGATGGC*

*AAAAGGGCCGTCGTGGGATACGAGGACGGCACCATCAGAATCTGGGACCTGAAGCAGGGCAGCCC*

*CATCCACGTGCTGAAGGGCACAGAAGGACACCAGGGCCCTCTGACATGCGTGGCCGCCAATCAGG*

*ATGGCAGCCTGATCCTGACCGGCAGCGTGGACTGTCAGGCCAAGCTGGTGTCTGCCACCACCGGC*

*AAAGTCGTGGGCGTGTTCAGACCCGAGACAGTGGCCAGCCAGCCTTCTCTGGGCGAGGGCGAGGA*

*ATCCGAGAGCAACAGCGTGGAAAACCCAGCTTTCTTGTACAAAGTGGTCCCC*

***AAMP (short variant)***

*GGGGACAAGTTTGTACAAAAAAGCAGGCTCCACCATGGACGAAGAGATCATCGAGGTGGTGGAACTG GACCCCGGACCCCCCGATCCTGATGATCTGGCCCAGGAAATGGAAGATGTGGACTTCGAGGAAGAAG AAGAGGAAGAGGGCAACGAAGAGGGCTGGGTGCTGGAACCTCAGGAAGGCGTCGTGGGCAGCATG GAAGGCCCCGACGATAGCGAAGTGACCTTCGCCCTGCACAGCGCCAGCGTGTTCTGCGTGTCCCTGGA CCCCAAGACCAACACCCTGGCTGTGACAGGCGGCGAGGACGACAAGGCTTTTGTGTGGCGGCTGAGC GACGGCGAGCTGCTGTTTGAATGTGCCGGCCACAAGGACAGCGTGACCTGCGCCGGCTTTAGCCACG ATAGCACCCTGGTGGCCACAGGCGATATGAGCGGCCTGCTGAAAGTGTGGCAGGTGGACACCAAAGA GGAAGTGTGGTCCTTCGAGGCCGGCGACCTGGAATGGATGGAATGGCACCCTAGAGCCCCCGTGCTG CTGGCCGGAACAGCCGATGGAAACACCTGGATGTGGAAGGTGCCCAACGGCGACTGCAAGACCTTCC AGGGCCCCAACTGTCCTGCCACCTGTGGCAGAGTGCTGCCCGATGGCAAAAGGGCCGTCGTGGGATA CGAGGACGGCACCATCAGAATCTGGGACCTGAAGCAGGGCAGCCCCATCCACGTGCTGAAGGGCACA GAAGGACACCAGGGCCCTCTGACATGCGTGGCCGCCAATCAGGATGGCAGCCTGATCCTGACCGGCA GCGTGGACTGTCAGGCCAAGCTGGTGTCTGCCACCACCGGCAAAGTCGTGGGCGTGTTCAGACCCGA GACAGTGGCCAGCCAGCCTTCTCTGGGCGAGGGCGAGGAAAGCGAGAGCAACAGCGTGGAAAACCC AGCTTTCTTGTACAAAGTGGTCCCC*

***CD164***

*GGGGACAAGTTTGTACAAAAAAGCAGGCTCCACCATGGACAAGAACACCACCCAGCACCCCAACGTG*

*ACCACCCTGGCCCCCATCAGCAATGTGACAAGCGCCCCTGTGACCAGCCTGCCCCTCGTGACAACACCA*

*GCCCCTGAGACATGCGAGGGCCGGAATAGCTGCGTGTCCTGCTTCAACGTGTCCGTCGTGAATACCAC*

*CTGTTTCTGGATCGAGTGCAAGGACGAGAGCTACTGCAGCCACAACAGCACCGTGTCCGACTGCCAAG*

*TGGGCAACACCACCGATTTCTGCAGCGTGTCCACCGCCACCCCTGTGCCTACCGCCAATAGCACCGCCA*

*AGCCTACCGTGCAGCCTAGCCCTAGCACCACCAGCAAGACCGTGACCACAAGCGGCACCACCAACAAC*

*ACAGTGACACCCACCAGCCAGCCCGTGCGGAAGTCCACCTTTGACAACCCAGCTTTCTTGTACAAAGTG*

*GTCCCC*

***KLRF1***

*GGGGACAAGTTTGTACAAAAAAGCAGGCTCCACCATGCTGCTGGTGTCCCAGGGCGTGCTGCTGAAG*

*TGTCAGAAGGGCAGCTGCAGCAACGCCACCCAGTACGAGGATACCGGCGACCTGAAAGTGAACAACG*

*GCACCCGGCGGAACATCAGCAACAAGGACCTGTGCGCCAGCCGCAGCGCCGATCAGACAGTGCTGTG*

*TCAGAGCGAGTGGCTGAAGTACCAGGGCAAGTGCTACTGGTTCAGCAACGAGATGAAGTCTTGGAGC*

*GACAGCTACGTGTACTGCCTGGAAAGAAAGAGCCATCTGCTGATCATCCACGACCAGCTGGAAATGGC*

*CTTCATCCAGAAGAACCTGCGGCAGCTGAACTACGTGTGGATCGGCCTGAACTTCACCAGCCTGAAGA*

*TGACCTGGACCTGGGTGGACGGCAGCCCTATCGACAGCAAGATCTTCTTCATCAAGGGCCCTGCCAAA*

*GAGAACAGCTGCGCCGCCATCAAAGAGTCCAAGATCTTTAGCGAGACATGCAGCAGCGTGTTCAAGT*

*GGATCTGCCAGTACAACCCAGCTTTCTTGTACAAAGTGGTCCCC*

***LILRB5 (variant 1)***

*GGGGACAAGTTTGTACAAAAAAGCAGGCTCCACCATGGGCACCCTGCCCAAGCCTACACTGTGGGCC*

*GAACCTGCCAGCGTGATCGCCAGAGGCAAGCCTGTGACCCTGTGGTGTCAGGGCCCCCTGGAAACCG*

*AGGAATACCGGCTGGACAAAGAGGGCCTGCCCTGGGCCAGAAAGAGACAGAACCCTCTGGAACCTG*

*GCGCCAAGGCCAAGTTCCACATCCCCAGCACCGTGTACGACAGCGCCGGCAGATACCGGTGCTACTAC*

*GAAACACCAGCCGGGTGGAGCGAGCCCAGCGATCCACTGGAACTGGTGGCCACAGGCTTCTACGCCG*

*AGCCAACACTGCTGGCCCTGCCTTCTCCTGTGGTGGCCTCTGGGGGCAACGTGACCCTGCAGTGCGAT*

*ACCCTGGATGGCCTGCTGACCTTCGTGCTGGTGGAAGAGGAACAGAAGCTGCCCCGGACCCTGTACA*

*GCCAGAAACTGCCTAAGGGCCCCAGCCAGGCCCTGTTTCCTGTGGGACCTGTGACACCCAGCTGCCGG*

*TGGCGGTTCAGATGTTACTACTACTACCGGAAGAACCCCCAAGTGTGGTCCAACCCCAGCGACCTGCT*

*GGAAATCCTGGTGCCCGGCGTGTCCAGAAAGCCCAGCCTGCTGATTCCTCAGGGCAGCGTGGTGGCT*

*AGAGGCGGCTCTCTGACTCTGCAGTGTAGAAGCGACGTGGGCTACGACATCTTTGTGCTGTACAAAGA*

*AGGCGAGCACGACCTGGTGCAGGGCTCCGGACAGCAGCCTCAGGCTGGACTGAGCCAGGCCAATTTC*

*ACACTGGGCCCCGTGTCTAGAAGCCACGGCGGCCAGTACAGATGCTACGGCGCCCACAATCTGAGCCC*

*CAGGTGGAGCGCACCTAGCGACCCCCTGGATATCCTGATCGCCGGCCTGATCCCCGACATCCCTGCTCT*

*GTCTGTGCAGCCTGGACCCAAGGTGGCCAGCGGCGAGAATGTGACACTGCTGTGCCAGAGCTGGCAC*

*CAGATCGACACATTCTTTCTGACAAAAGAGGGCGCAGCCCACCCCCCACTGTGCCTGAAGTCTAAGTAC*

*CAGAGCTACCGGCACCAGGCCGAGTTCAGCATGAGCCCTGTGACATCTGCCCAGGGCGGCACCTACA*

*GGTGCTACTCCGCCATCAGAAGCTACCCCTACCTGCTGAGCAGCCCCAGCTACCCCCAGGAACTGGTG*

*GTGTCTGGCCCTAGCGGAGATCCTAGCCTGAGCCCTACCGGCAGCACACCTACACCAGCTGGCCCCGA*

*AGATCAGCCCCTGACCCCTACTGGACTGGACCCTCAGAGTGGCCTGGGAAGGCATCTGGGAAACCCA*

*GCTTTCTTGTACAAAGTGGTCCCC*

***LILRB5 (variant 2)***

*GGGGACAAGTTTGTACAAAAAAGCAGGCTCCACCATGGGCACCCTGCCCAAGCCTACACTGTGGGCC*

*GAACCTGCCAGCGTGATCGCCAGAGGCAAGCCTGTGACCCTGTGGTGTCAGGGCCCCCTGGAAACCG*

*AGGAATACCGGCTGGACAAAGAGGGCCTGCCCTGGGCCAGAAAGAGACAGAACCCTCTGGAACCTG*

*GCGCCAAGGCCAAGTTCCACATCCCCAGCACCGTGTACGACAGCGCCGGCAGATACCGGTGCTACTAC*

*GAAACACCAGCCGGGTGGAGCGAGCCCAGCGATCCACTGGAACTGGTGGCTACCGGCGTGTCCAGAA*

*AGCCCAGCCTGCTGATCCCTCAGGGCAGCGTGGTGGCTAGAGGCGGCTCTCTGACCCTGCAGTGCAG*

*ATCCGACGTGGGCTACGACATCTTCGTGCTGTACAAAGAAGGCGAGCACGACCTGGTGCAGGGCTCC*

*GGACAGCAGCCTCAGGCTGGACTGAGCCAGGCCAATTTCACACTGGGCCCCGTGTCTAGAAGCCACG*

*GCGGCCAGTACAGATGCTACGGCGCCCACAATCTGAGCCCCAGGTGGAGCGCACCTAGCGACCCCCT*

*GGATATCCTGATCGCCGGCCTGATCCCCGACATCCCTGCTCTGTCTGTGCAGCCTGGACCCAAGGTGGC*

*CAGCGGCGAGAATGTGACACTGCTGTGCCAGAGCTGGCACCAGATCGACACATTCTTTCTGACAAAAG*

*AGGGCGCAGCCCACCCCCCACTGTGCCTGAAGTCTAAGTACCAGAGCTACCGGCACCAGGCCGAGTTC*

*AGCATGAGCCCTGTGACATCTGCCCAGGGCGGCACCTACAGGTGCTACTCCGCCATCAGAAGCTACCC*

*CTACCTGCTGAGCAGCCCCAGCTACCCCCAGGAACTGGTGGTGTCTGGCCCTAGCGGAGATCCTAGCC*

*TGAGCCCTACCGGCAGCACACCTACACCAGCTGGCCCCGAAGATCAGCCCCTGACCCCTACTGGACTG*

*GACCCTCAGAGTGGCCTGGGAAGGCATCTGGGAAACCCAGCTTTCTTGTACAAAGTGGTCCCC*

***OLR1 (variant 1)***

*GGGGACAAGTTTGTACAAAAAAGCAGGCTCCACCATGCAGCTGAGCCAGGTGTCCGATCTGCTGACCC*

*AGGAACAGGCCAACCTGACCCACCAGAAGAAGAAGCTGGAAGGCCAGATCAGCGCCAGACAGCAGG*

*CCGAGGAAGCCAGCCAGGAAAGCGAGAACGAGCTGAAAGAGATGATCGAGACACTGGCCCGGAAGC*

*TGAACGAGAAGTCCAAAGAACAGATGGAACTGCACCACCAGAACCTGAACCTGCAGGAAACCCTGAA*

*GCGGGTGGCCAACTGCAGCGCCCCATGTCCCCAGGATTGGATCTGGCACGGCGAGAACTGCTACCTGT*

*TCAGCAGCGGCAGCTTCAACTGGGAGAAGTCCCAGGAAAAGTGCCTGAGCCTGGACGCCAAGCTGCT*

*GAAGATCAACAGCACCGCCGACCTGGACTTCATACAGCAGGCTATCAGCTACAGCAGCTTCCCCTTCTG*

*GATGGGCCTGAGCAGACGGAACCCCAGCTACCCTTGGCTGTGGGAGGATGGCAGCCCCCTGATGCCC*

*CACCTGTTTAGAGTGCGGGGAGCCGTGTCCCAGACCTACCCTAGCGGAACCTGCGCCTACATCCAGAG*

*GGGCGCTGTGTACGCCGAGAATTGCATCCTGGCCGCCTTCAGCATCTGTCAGAAGAAGGCCAATCTGC*

*GGGCCCAGAACCCAGCTTTCTTGTACAAAGTGGTCCCC*

***OLR1 (variant 2)***

*GGGGACAAGTTTGTACAAAAAAGCAGGCTCCACCATGCAGCTGAGCCAGGTGTCCGATCTGCTGACCC*

*AGGAACAGGCCAACCTGACCCACCAGAAGAAGAAGCTGGAAGGCCAGATCAGCGCCAGACAGCAGG*

*CCGAGGAAGCCAGCCAGGAAAGCGAGAACGAGCTGAAAGAGATGATCGAGACACTGGCCCGGAAGC*

*TGAACGAGAAGTCCAAAGAACAGATGGAACTGCACCACCAGAACCTGAACCTGCAGGAAACCCTGAA*

*GCGGGTGGCCAACTGTAGCGGACTGCACCCCGCCAGCAACTTTCTGTTCCAGTTCAGCATCCTGGATG*

*GCGCCGTGTCCGAGGAACCCCAGCTGCCTATGGCTCTGGGCGGCAGATTCAGCTTCGACGCCCCTCTG*

*ATCAACCCAGCTTTCTTGTACAAAGTGGTCCCC*

***OLR1 (variant 3)***

*GGGGACAAGTTTGTACAAAAAAGCAGGCTCCACCATGCAGCTGAGCCAGGTGTCCGATCTGCTGACCC*

*AGGAACAGGCCAACCTGACCCACCAGAAGAAGAAGCTGGAAGGCCAGATCAGCGCCAGACAGCAGG*

*CCGAGGAAGCCAGCCAGGAAAGCGAGAACGAGCTGAAAGAGATGATCGAGACACTGGCCCGGAAGC*

*TGAACGAGAAGTCCAAAGAACAGATGGAACTGCACCACCAGAACCTGAACCTGCAGGAAACCCTGAA*

*GCGGGTGGCCAACTGCAGCGCCCCATGTCCCCAGGATTGGATCTGGCACGGCGAGAACTGCTACCTGT*

*TCAGCAGCGGCAGCTTCAACTGGGAGAAGTCCCAGGAAAAGTGCCTGAGCCTGGACGCCAAGCTGCT*

*GAAGATCAACAGCACCGCCGACCTGATCAACCCAGCTTTCTTGTACAAAGTGGTCCCC*

***PAG1***

*GGGGACAAGTTTGTACAAAAAAGCAGGCTCCACCATGGGCCCAGCCGGCAGTCTGCTGGGATCTGGA*

*CAGATGCAGATCACCCTGTGGAACCCAGCTTTCTTGTACAAAGTGGTCCCC*

*PTCA GGGGACAAGTTTGTACAAAAAAGCAGGCTCCACCATGGCCCTGCCCTGCACACTGGGACTGGGAATG*

*CTGCTGGCTCTGCCTGGCGCTCTGGGATCTGGCGGATCTGCCGAGGATAGCGTGGGCAGCAGTAGCA*

*ACCCAGCTTTCTTGTACAAAGTGGTCCCC*

***PTPRA***

*GGGGACAAGTTTGTACAAAAAAGCAGGCTCCACCATGAACAACGCCACCACCGTGGCCCCTAGCGTG*

*GGCATCACCAGACTGATCAACAGCAGCACCGCCGAGCCCGTGAAAGAGGAAGCCAAGACCAGCAACC*

*CCACCAGCAGCCTGACAAGCCTGAGCGTGGCCCCCACCTTCAGCCCCAATATCACACTGGGCCCCACCT*

*ACCTGACCACCGTGAACAGCAGCGACAGCGACAATGGCACCACCAGAACCGCCAGCACCAACAGCAT*

*CGGCATCACAATCTCCCCCAACGGCACCTGGCTGCCCGACAACCAGTTCACCGATGCCAGAACCGAGC*

*CCTGGGAGGGCAATAGCTCTACCGCCGCCACCACACCCGAGACATTTCCACCCAGCGGCAACAGCGAC*

*AACCCAGCTTTCTTGTACAAAGTGGTCCCC*

***PTPRC***

*GGGGACAAGTTTGTACAAAAAAGCAGGCTCCACCATGCAGAGCCCCACCCCTTCTCCAACCGGCCTGA*

*CCACAGCCAAGATGCCTAGCGTGCCCCTGAGCAGCGATCCCCTGCCTACACACACCACCGCCTTTAGCC*

*CTGCCAGCACCTTCGAGAGAGAGAACGACTTCAGCGAGACAACCACCAGCCTGAGCCCCGACAACACC*

*AGCACACAGGTGTCCCCCGACAGCCTGGATAACGCCAGCGCCTTCAATACCACCGGCGTGTCCAGCGT*

*GCAGACCCCCCATCTGCCAACACACGCCGATAGCCAGACACCTAGCGCCGGCACCGATACCCAGACCT*

*TTTCTGGCTCTGCCGCCAACGCCAAGCTGAACCCTACCCCTGGCAGCAACGCCATCTCCGATGTGCCTG*

*GCGAGAGAAGCACCGCCTCCACCTTTCCAACCGACCCCGTGTCTCCTCTGACCACCACACTGTCTCTGG*

*CCCACCACAGCTCTGCTGCCCTGCCTGCCAGAACCAGCAACACCACCATCACCGCCAACACCTCCGACG*

*CCTACCTGAACGCCTCCGAAACCACAACCCTGTCCCCATCTGGCAGCGCCGTGATCAGCACCACCACAA*

*TCGCCACCACCCCCAGCAAGCCTACCTGCGACGAGAAGTACGCCAACATCACCGTGGACTACCTGTAC*

*AACAAAGAGACAAAGCTGTTCACCGCTAAGCTGAACGTGAACGAGAACGTGGAATGCGGCAACAATA*

*CCTGCACCAACAACGAGGTGCACAACCTGACCGAGTGCAAGAATGCCAGCGTGTCCATCAGCCACAAC*

*AGCTGCACCGCCCCTGACAAGACCCTGATCCTGGATGTGCCCCCTGGCGTGGAAAAGTTCCAGCTGCA*

*CGACTGCACCCAGGTGGAAAAGGCCGATACCACCATCTGCCTGAAGTGGAAGAACATCGAGACATTC*

*ACCTGTGACACCCAGAACATCACCTACCGGTTCCAGTGCGGCAATATGATCTTCGATAACAAAGAAATC*

*AAGCTGGAAAACCTGGAACCCGAGCACGAGTACAAGTGCGACAGCGAGATCCTGTATAACAACCACA*

*AGTTCACCAACGCCAGCAAGATCATCAAGACCGACTTCGGCAGCCCTGGCGAGCCCCAGATCATCTTCT*

*GTAGAAGCGAGGCCGCCCACCAGGGCGTGATCACCTGGAATCCTCCCCAGCGGAGCTTCCACAACTTC*

*ACCCTGTGCTACATCAAAGAAACCGAGAAGGACTGCCTGAACCTGGACAAGAATCTGATTAAGTACGA*

*CCTGCAGAACCTGAAGCCCTACACCAAATACGTGCTGAGCCTGCACGCCTACATCATTGCCAAGGTGC*

*AGCGGAATGGAAGCGCCGCCATGTGCCACTTCACCACCAAGAGCGCCCCTCCCAGCCAAGTGTGGAAC*

*ATGACCGTGTCCATGACCAGCGACAACTCCATGCACGTGAAGTGCCGGCCTCCCCGGGACAGAAATGG*

*CCCCCACGAGAGATACCACCTGGAAGTGGAAGCCGGCAACACCCTCGTGCGGAACGAGAGCCACAAG*

*AACTGCGACTTCAGAGTGAAGGACCTGCAGTACAGCACCGACTACACCTTCAAGGCCTACTTCCACAAT*

*GGCGACTACCCCGGCGAGCCCTTCATCCTGCACCACAGCACCAGCTACAACAGCAAGAACCCAGCTTT*

*CTTGTACAAAGTGGTCCCC*

***SLAMF6 (variant 1)***

*GGGGACAAGTTTGTACAAAAAAGCAGGCTCCACCATGCAGAGCAGCCTGACCCCCCTGATGGTCAAC*

*GGCATCCTGGGCGAGAGCGTGACCCTGCCCCTGGAATTTCCTGCCGGCGAGAAAGTGAACTTCATCAC*

*CTGGCTGTTCAACGAGACAAGCCTGGCCTTCATCGTGCCCCACGAGACAAAGTCCCCCGAGATCCACG*

*TGACCAACCCCAAGCAGGGCAAGCGGCTGAACTTCACCCAGAGCTACAGCCTGCAGCTGAGCAACCTG*

*AAGATGGAAGATACCGGCAGCTACCGGGCCCAGATCAGCACCAAGACAAGCGCCAAGCTGAGCAGCT*

*ACACCCTGCGGATCCTGCGGCAGCTGCGGAATATCCAAGTGACAAACCACAGCCAGCTGTTCCAGAAC*

*ATGACCTGCGAGCTGCACCTGACCTGCAGCGTGGAAGATGCCGACGACAACGTGTCCTTCAGATGGG*

*AGGCCCTGGGCAACACCCTGAGCAGCCAGCCTAATCTGACCGTGTCCTGGGACCCCCGGATCAGCAGC*

*GAGCAGGACTACACCTGTATCGCCGAGAACGCCGTGTCCAACCTGAGCTTCTCCGTGTCCGCCCAGAA*

*ACTGTGCGAGGACGTGAAGATCCAGTACACCGACACCAAGATGAACCCAGCTTTCTTGTACAAAGTGG*

*TCCCC*

***SLAMF6 (variant 2)***

*GGGGACAAGTTTGTACAAAAAAGCAGGCTCCACCATGCAGCTGCGGAACATCCAAGTGACCAACCAC*

*AGCCAGCTGTTCCAGAACATGACCTGCGAGCTGCACCTGACCTGCAGCGTGGAAGATGCCGACGACA*

*ACGTGTCCTTCAGATGGGAGGCCCTGGGCAACACCCTGAGCAGCCAGCCTAATCTGACCGTGTCCTGG*

*GACCCCCGGATCAGCAGCGAGCAGGACTACACCTGTATCGCCGAGAACGCCGTGTCCAACCTGAGCTT*

*CTCCGTGTCCGCCCAGAAACTGTGCGAGGACGTGAAGATCCAGTACACCGACACCAAGATGAACCCAG*

*CTTTCTTGTACAAAGTGGTCCCC*

***SLAMF7 (variant 1)***

*GGGGACAAGTTTGTACAAAAAAGCAGGCTCCACCATGAGCGGCCCTGTGAAAGAACTCGTGGGCTCT*

*GTGGGCGGAGCCGTGACCTTTCCACTGAAGTCCAAAGTGAAACAGGTGGACAGCATCGTGTGGACCT*

*TCAACACCACCCCCCTCGTGACCATCCAGCCTGAGGGCGGCACCATCATCGTGACCCAGAACCGGAAC*

*CGCGAGCGGGTGGACTTTCCTGATGGCGGCTACAGCCTGAAGCTGAGCAAGCTGAAGAAGAACGACA*

*GCGGCATCTACTACGTGGGCATCTACAGCAGCAGCCTGCAGCAGCCCTCCACCCAGGAATACGTGCTG*

*CACGTGTACGAGCACCTGTCCAAGCCCAAAGTGACCATGGGCCTGCAGAGCAACAAGAACGGCACCT*

*GTGTGACCAACCTGACCTGCTGCATGGAACACGGCGAAGAGGACGTGATCTACACCTGGAAGGCCCT*

*GGGCCAGGCCGCCAACGAAAGCCACAATGGCAGCATCCTGCCCATCTCTTGGAGATGGGGCGAGAGC*

*GACATGACCTTCATCTGCGTGGCCCGGAACCCCGTGTCCCGGAACTTTAGCTCTCCCATCCTGGCCAGA*

*AAGCTGTGCGAGGGCGCTGCCGACGATCCCGATAGCTCTATGAACCCAGCTTTCTTGTACAAAGTGGT*

*CCCC*

***SLAMF7 (variant 2)***

*GGGGACAAGTTTGTACAAAAAAGCAGGCTCCACCATGGGCAGCGCCGCCTCTGGACCTGTGAAAGAA*

*CTCGTGGGCTCTGTGGGCGGAGCCGTGACCTTTCCACTGAAGTCCAAAGTGAAACAGGTGGACAGCA*

*TCGTGTGGACCTTCAACACCACCCCCCTCGTGACCATCCAGCCTGAGGGCGGCACCATCATCGTGACCC*

*AGAACCGGAACCGCGAGCGGGTGGACTTTCCTGATGGCGGCTACAGCCTGAAGCTGAGCAAGCTGAA*

*GAAGAACGACAGCGGCATCTACTACGTGGGCATCTACAGCAGCAGCCTGCAGCAGCCCTCCACCCAG*

*GAAAACCCAGCTTTCTTGTACAAAGTGGTCCCC*
